# Right ventricule-specific therapies in ARDS: other vasodilating agents to be considered

**DOI:** 10.1186/s13054-023-04419-4

**Published:** 2023-04-03

**Authors:** Senada Ymeraj, Rachid Attou, Sebastien Redant

**Affiliations:** 1 Departement of Anesthesiology, Erasme Hospital, Université Libre de Bruxelles (ULB), Route de Lennik 808, 1070 Brussels, Belgium; 2 Departement of Intensive Care, Brugmann Hospital, Université Libre de Bruxelles (ULB), Place Van Gehuchten 4, 1090 Brussels, Belgium

We read with great attention the Ganeriwal et al.’s review on useful treatments for the right heart due to acute respiratory distress syndrome [1]. We saw that the authors did not mention milrinone. Milrinone is a phosphodiesterase inhibitor and “inodilator” that increases contractility and RV relaxation. It decreases pulmonary as well as systemic resistance but may worsen existing hypotension [2].

We also read in a study by Morelli et al.’s, a 24-h infusion of levosimendan, another inodilator, improved contractility and decreased pulmonary resistance (compared to a placebo) in 35 patients with pulmonary hypertension upon the onset of ARDS and septic shock [3]. However, this drug needs further investigation in its capacity to treat ARDS due to its risks of arrhythmias and systemic hypotension.

In another study, 10 patients with ARDS were given one 50 mg dose of sildenafil. Patients showed a significant decrease in pulmonary hypertension and the right ventricle afterload. However, patients also experienced detrimental effects, such as an increase in the intrapulmonary shunt, a decrease in PaO_2_ and systemic arterial pressure. Due to these adverse effects, the authors did not recommend the systematic use of sildenafil in treating ARDS [4].

Emphasis must be placed on the fact that many of these IV or PO drugs derive from essential pulmonary arterial hypertension treatments, and they all suffer from the same two problems. First, a lack of pulmonary selectivity, inducing a concomitant systemic vasodilation that can be harmful if it’s associated with hemodynamic instability. Second, these vasodilators act on all the vessels of the lung, in both ventilated and non-ventilated areas by increasing the intrapulmonary shunt and reducing PaO_2_ [5]. While this is not the case for inhaled NO, which acts only in the ventilated lung where it gets degraded and has no effect on systemic blood pressure, it doesn’t change mortality [5].

## Data Availability

Not applicable.

## References

[1] Ganeriwal S, Alves dos Anjos G, Schleicher M (2023). Right ventricle-specific therapies in acute respiratory distress syndrome: a scoping review. Crit Care.

[2] Chen EP, Bittner HB, Davis RD, Van Trigt P (1997). Milrinone improves pulmonary hemodynamics and right ventricular function in chronic pulmonary hypertension. Ann Thorac Surg.

[3] Morelli A, Teboul JL, Maggiore SM, Vieillard-Baron A, Rocco M, Conti G, De Gaetano A, Picchini U, Orecchioni A, Carbone I, Tritapepe L, Pietropaoli P, Westphal M (2006). Effects of levosimendan on right ventricular afterload in patients with acute respiratory distress syndrome: a pilot study. Crit Care Med.

[4] Cornet AD, Hofstra JJ, Swart EL, Girbes AR, Juffermans NP (2010). Sildenafil attenuates pulmonary arterial pressure but does not improve oxygenation during ARDS. Intensive Care Med.

[5] Moloney ED, Evans TW (2003). Pathophysiology and pharmacological treatment of pulmonary hypertension in acute respiratory distress syndrome. Eur Respir J.

